# Sensory integration and activities of daily living in children with developmental coordination disorder

**DOI:** 10.1186/1824-7288-38-14

**Published:** 2012-07-12

**Authors:** Bülent Elbasan, Hlya Kayıhan, Irem Duzgun

**Affiliations:** 1 Gazi University, Faculty of Health Sciences, Department of Physiotherapy and Rehabilitation, Emniyet mh. Muammer Yaşar Bostancı cd. No: 14 Beşevler, Ankara, Turkey; 2 Hacettepe University, Faculty of Health Sciences, Department of Ergotherapy, Ankara, Turkey

**Keywords:** Developmental coordination disorder, Sensory integration, Activities of daily living

## Abstract

**Objective:**

The aim of our study was to evaluate sensory integration and activities of daily living in children with developmental coordination disorder

**Subjects and methods:**

37 cases with developmental coordination disorder and 35 healthy age-matched peers were included in this study. Ayres Southern California Sensory Integration Test was used for evaluating the sensory integration and Functional Independence Measure for Children (WeeFIM) was used for evaluating the activities of daily living.

**Results:**

Significant differences were found in the visual shape perception, position in space, and design copying (*p* < 0.05). According to the results of somatosensory perception tests, significant differences were found in kinesthesia, manual form perception, finger identification, figure-ground perception, localization of tactile stimuli, double tactile stimuli perception (*p* < 0.05). Control group was better in motor planning (*p* < 0.05). Comprehension, expression, social communication, problem solving, and memory skills were significant in favor of the control group (*p* < 0.05). Graphestesia and self-care domain was found to be correlated (r = 0,491, *p* = 0.002) between the groups.

**Discussion:**

Special education and rehabilitation programs including sensory integration therapy and motor performance will increase independence in the activities of daily living in children with developmental coordination disorder.

## Introduction

Developmental coordination disorder (DCD) was described as “impairment or immaturity of the organization of the movement” by Dyspraxia Foundation [1]. Children with DCD may display a wide range of motor problems including delays in accomplishing motor milestones such as walking and sitting, dropping things, and poor performance in sports or in handwriting [2]. Although not involved in any classification system, most commonly used names are “clumsy child syndrome”, “the original developmental disorder of the motor functions” as defined in ICD-10, and the “developmental coordination disorder” as defined in DSM-IV. This term is accepted by American Psychiatric Association (APA) in 1994 [3].

Some symptoms of DCD may vary with age. Delays in motor development in young children such as sitting, crawling and walking, and difficulties in self-dressing and eating may be seen. Balance problems, clumsiness, frequent fallings and injuries may occur in pre-school periods and also incompetence in cycling, throwing and catching a ball, and difficulties in jumping and balance can be seen [4,5]. Overall, children with Developmental Coordination Disorder showed an impaired ability to produce familiar gestures compared to their typical peers, and this was dependant on the type of gesture and presentation modality [6].

Ayres described DCD as a sensory integration disorder. This information was achieved by the results of several factor analyze studies which were done to investigate the scores between the tactile tests and motor planning tests [7,8].

There are many aspects of DCD and its management. Thirty percent of school-aged children were shown to have a sensory integration disorder in different studies [9-11]. Sensory integration is one of the aspects and should be assessed in details. It may affect the development of coordination which may lead to deficiencies in activities of daily living. But the deficiencies in the activities are uncertain yet.

In the literature, children with developmental coordination disorder are seen to be huge in numbers, but research findings are limited for rehabilitation approaches. Our study was planned to determine the relationships between sensory integration and activities of daily living in children with DCD. This will help to establish more effective rehabilitation programs for these children.

## Subjects and method

**Figure 1:**
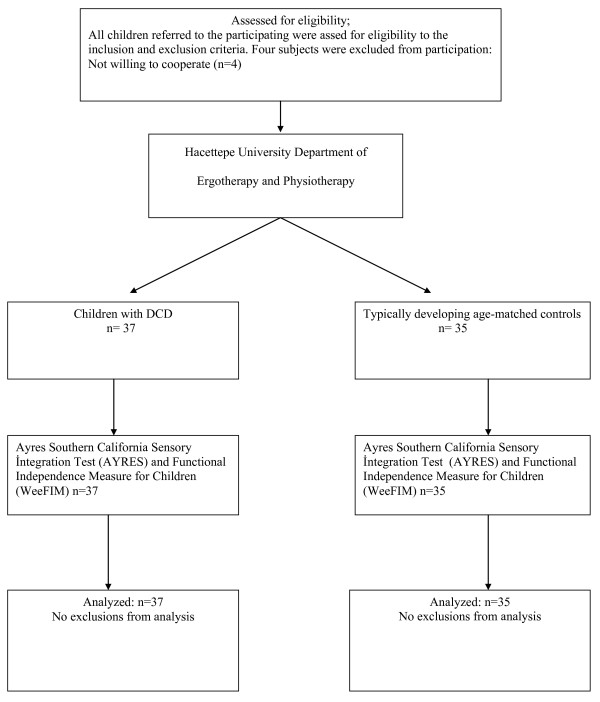
Standard flow chart of the study.

Thirty-seven children (16 girls and 21 boys) with DCD were referred by a single pediatric psychiatrist and 35 (17 girls and 18 boys) typically developing children who served as age-matched controls, evaluated at Hacettepe University, were enrolled in this study. The design of the study is given in a standard flow chart (Figure 1).

The criteria for participation in the study included; age between 9–10 years at the time of intake; previous identification by a qualified physician as having the diagnosis of developmental coordination disorder; normal intelligence; normal hearing and vision; and child and parental consent and agreement to participate. Children who met the following criteria were excluded from participation in the study; previous or present exposure to a cognitive-based treatment for motor problems; and medical diagnosis of a specific neurological disorder or a physical or sensory deficit causing the motor problem.

All subjects read and gave written informed constent on a university approved constent form, from the Ethics Committee of the Hacettepe University (LUT 09/48).

All evaluations were done by a single physiotherapist qualified in pediatrics who had 12 years of experience and was blinded to group membership. Patients and parents were informed about the evaluation procedure and the outcomes. All evaluations were done in an isolated room one by one.

Ayres Southern California Sensory Integration Test was used for evaluating the sensory integration, and the Functional Independence Measure for Children (WeeFIM) was used for evaluating the activities of daily living.

Ayres Southern California Sensory Integration Tests is a standardized test used worldwide for assessment of the sensory integration, and was developed in 1980 by Jean Ayres. The purpose of the tests is to find out and define the inadequate functions in the sensory integration. These tests evaluate the visual perception, somatosensory perception, and motor performance with the body mid-line crossing and right-left discrimination [12].

WeeFIM was developed by a team to identify the neurodevelopmental deficiencies, to meet the needs for fully evaluating these disabilities, and to assess the results of biomedical, developmental, and psychosocial interventions. Functional Independence Measure for Children (WeeFIM) was derived from Functional Independence Measure (FIM) that developed for adults by Uniform Data System for Medical Rehabilitation (UDS) [13]. WeeFIM measures the functional independence in children. Two approaches that related to functional independence constitutes the basis for WeeFIM [14]. WeeFIM can be used in children between 6 months and 12 years and with developmental disorders, in children of any age with a mental age below 7, and in children between 6 months and 8 years without any disorders [15]. It evaluates the fields of personal care, movement skills, and perception with the sub-parameters.

## Statistical analysis

SPSS for Windows 15.0 software package was used for the statistical analyses of this study. Differences of the results of Ayres Southern California Sensory Integration Test, and WeeFIM between the cases and the control group were analyzed with *t* test.

Relationships between Ayres Southern California Sensory Integration Test and WeeFIM were evaluated by pearson correlation analysis. P-values less than 0.05 were considered statistically significant.

## Results

Mean ages (± Standard Deviations) of the 21 boys and 16 girls in the study group, and 17 boys and 18 girls in the control group were 10 (±1.5) years and 10 (±2) years, 10 (±0.8) years and 9 (±1) years, respectively. There were no statistically significant differences between the ages of the groups (*p* > 0.05).

Visual perception and somatosensory perception test results of Ayres Southern California Sensory Integration Test of the groups were shown in Table 1, and motor performance and other test results are shown in Table 2.

**Table 1:** Ayres visual and somatosensory perception test results

	**Space Visualization X ± SD**	**Figure- Ground Perception X ± SD**	**Position in space X ± SD**	**Design copying X ± SD**	**Kinesthesia X ± SD**	**Manual form perception X ± SD**	**Finger identification X ± SD**	**Graphesthesia X ± SD**	**Localization of tactile stimuli X ± SD**	**Double tactile stimuli perception X ± SD**
**Group 1**	18.05 ± 6.45	24 ± 5.33	14.89 ± 5.41	16.14 ± 4.82	88.22 ± 5.96	8.38 ± 2.81	10 ± 5.63	12.95 ± 3.13	80.92 ± 15.62	20.24 ± 9.99
**Group 2**	24.34 ± 2.9	25.29 ± 3.18	18.6 ± 3.5	19.06 ± 4.6	85.37 ± 4.67	11.06 ± 0.68	14.46 ± 2.62	14.63 ± 3.63	94.04 ± 4.05	29.34 ± 3.12
**p**	0.000	0.222	0.001	0.010	0.028	0.000	0.000	0.039	0.000	0.000

Group 1: Children with DCD, Group 2: Typically developing children, *X* mean value, *SD* Standard deviation, (*p* < 0.05).

**Table 2:** Ayres motor performance and other test results

	**İmitation of postures X ± SD**	**Bilateral motor coordination X ± SD**	**Motor accuracy X ± SD**	**Left-right discrimination X ± SD**	**Crossing midline of body X ± SD**
**Group 1**	19.43 ± 3.4	12.16 ± 3.9	133.4 ± 15.4	15 ± 4.3	23.14 ± 2.2
**Group 2**	21.89 ± 1.6	15.06 ± 1.2	107.4 ± 23.2	18.23 ± 2.5	23.91 ± 0.3
**p**	0.000	0.000	0.000	0.000	0.043
**t**	−3.875	−4.142	5.480	−3.844	−2.066

Group 1: Children with DCD, Group 2: Typically developing children, *X* mean value, *SD* Standard deviation, (*p* < 0.05).

Significant differences were found in the visual shape perception, position in space, and design copying (*p* < 0.05). According to the results of somatosensory perception tests, significant differences were found in kinesthesia, manual form perception, finger identification, figure-ground perception, localization of tactile stimuli, double tactile stimuli perception (*p* < 0.05). Statistically significant differences were found in imitation of postures, bilateral motor coordination, and motor accuracy tests (*p* < 0.05).

Results of the self care test which is used to evaluate the activities of daily living are shown in Table 3. Results of mobility tests and perception tests are shown at Table 4.

**Table 3:** WeeFIM self-care test results

	**Eating X ± SD**	**Grooming X ± SD**	**Bathing X ± SD**	**Dressing upper body X ± SD**	**Dressing lower body X ± SD**	**Toiletting X ± SD**	**Bladder management X ± SD**	**Bowel management X ± SD**
**Group 1**	7 ± 0	5.97 ± 0.928	5.84 ± 0.898	6.81 ± 0.569	6.84 ± 0.553	6.7 ± 0.702	7 ± 0	7 ± 0
**Group 2**	7 ± 0	7 ± 0	7 ± 0	7 ± 0	7 ± 0	7 ± 0	7 ± 0	7 ± 0
**p**	1.000	0.000	0.000	0.053	0.088	0.015	1.000	1.000
**t**	1.000	−6.548	−7.654	−1.965	−1.733	−2.505	1.000	1.000

Group 1: Children with DCD, Group 2: Typically developing children, *X* mean value, *SD* Standard deviation, (*p* < 0.05).

**Table 4:** WeeFIM mobility and perception test results

	**Wheelchair/ chair transfer X ± SD**	**Toilet transfer X ± SD**	**Bath/ shower transfer X ± SD**	**Crawling walking, wheelchair X ± SD**	**Stair climbing X ± SD**	**Comprehension X ± SD**	**Expression X ± SD**	**Social interaction X ± SD**	**Problem solving X ± SD**	**Memory X ± SD**
**Group 1**	7 ± 0	7 ± 0	7 ± 0	7 ± 0	7 ± 0	5.72 ± 1.233	5.19 ± 1.221	5.78 ± 1.228	4.76 ± 0.955	4.62 ± 0.982
**Group 2**	7 ± 0	7 ± 0	7 ± 0	7 ± 0	7 ± 0	6.54 ± 0.505	6.8 ± 0.473	6.71 ± 0.519	6.57 ± 0.558	6.66 ± 0.539
**p**	1.000	1.000	1.000	1.000	1.000	0.001	0.000	0.000	0.000	0.000
**t**	1.000	1.000	1.000	1.000	1.000	−3.650	−7.302	−4.146	−9.776	−9.590

Group 1: Children with DCD, Group 2: Typically developing children, *X* mean value, *SD* Standard deviation, (*p* < 0.05).

Both groups were at the same level in most of the self care domains. Comprehension, expression, social communication, problem solving, and memory skills were significant in favor of the control group (*p* < 0.05). Graphestesia and self-care domain was found to be correlated (r = 0,491, *p* = 0.002) between the groups.

When the correlation between the Ayres Southern California Sensory Integration Tests and WeeFIM parameters were checked, correlation was found between graphestesia and self-care in the assessments of the children with neurodevelopmental disorders (r: 0.491, *p*: 0.002). For the control group, correlation was found between kinesthesia and social communication (r: 0.376, *p*: 0.026), and imitation of postures and social communication (r: 0.393, *p*: 0.02) (*p* < 0.05) (Table 5).

**Table 5:** Correlation between Ayres Sensory Integration Test and WeeFIM

	**Group**	**Self Care**		**Communication**		**Social Interaction**	
		**r**	**p**	**r**	**p**	**r**	**p**
**Visual Shape Perception**	1	−0.069	0.685	0.256	0.125	0.110	0.518
	2			0.236	0.172	0.178	0.306
**Figure Ground Perception**	1	0.025	0.882	−0.023	0.892	0.16 0	0.343
	2			−0.05	0.778	−0.257	0.136
**Spatial Perception**	1	0.065	0.702	0.299	0.073	0.106	0.532
	2			0.115	0.509	0.035	0.840
**Kinesthesia**	1	0.226	0.179	0.125	0.461	0.203	0.229
	2			−0.103	0.556	−0.376	0.026*
**Hand Shape Recognition**	1	0.040	0.814	0.101	0.551	0.050	0.767
	2			0.021	0.904	0.143	0.411
**Finger Identification**	1	0.153	0.365	0.251	0.134	0.157	0.352
	2			−0.079	0.654	−0.037	0.833
**Graphesthesia**	1	0.491	0.002*	0.32	0.053	0.004	0.980
	2			0.069	0.693	−0.202	0.245
**Localization of Tactile Stimuli**	1	−0.095	0.577	−0.008	0.962	−0.153	0.367
	2			0.318	0.063	0.168	0.334
**Localization of Double Tactile Stimuli**	1	−0.096	0.574	0.236	0.160	0.161	0.343
	2			−0.105	0.549	0.135	0.440
**Postural Imitation**	1	0.049	0.772	0.228	0.176	0.010	0.909
	2			0.181	0.299	0.393	0.020*
**Midline Crossing**	1	−0.064	0.705	0.173	0.305	0.075	0.657
	2			−0.157	0.366	−0.182	0.295
**Bilateral Motor Coordination**	1	0.104	0.540	−0.021	0.901	−0.176	0.296
	2			0.211	0.225	0.105	0.549
**Left-Right Discrimination**	1	−0.078	0.647	0.184	0.275	−0.038	0.822
	2			0.187	0.282	0.080	0.648

Group 1: Children with DCD, Group 2: Typically developing children.

## Discussion

It is shown that the problems in taking visual, tactile and proprioceptive inputs and integrating them in an appropriate way leads to deficiencies in activities of daily living in children with DCD. Tactile and proprioceptive contents should constitute an important role in the rehabilitation programme of children with developmental coordination disorder to be more independent in the activities of daily living.

When the literature is reviewed, it can be seen that the children with developmental coordination disorders are relatively huge in numbers, but the study findings for the rehabilitation approaches are limited. In limited number of studies motor problems were associated with some parameters of sensory integration, but have not been compared with the independence in activities of daily living.

By using Ayres Southern California Sensory Integration Test in our study, it was seen that except figure ground perception, the children with DCD were inadequate at the level of sensory processing according to their healthy peers. Deficiency in motor planning, poor motor coordination, lack of the process in tactile, proprioceptive and kinesthetic inputs are common problems in children with DCD and they demonstrate a heavy reliance on visual feedback to guide task performance [16-18]. According to these problems, these children may have difficulties in sensory integration process. [7,8]. It is taught that, position in space, space visualization and design copying parameters are affected adversely because of that reason.

More recent research suggests that in children with DCD, visual feedback is managed differently and processed more slowly than in typically developing children [19,20]. Also this result is thought to be caused from the education program which the children continue to receive. Persisting of sensory integration problems, although they are receiving education, reveals the need for the implementation of specific education and treatment of this issue.

In some studies it is shown that at the age of 8,4 the children with DCD present the same performance in figure ground perception compared with their healthy peers [21]. It is related that, children with DCD were as able as control children to detect figures when they are hidden in a complex confusing background [21]. In the studies done by Lord and Hulme, they concluded that there was a difference in figure ground perception at the age of 9.8 between the children with DCD and their healthy peers [22,23]. It is taught that development in visual perception in healthy children is faster than in children with DCD. Although, it is concluded that the development in figure ground perception in healthy children is more evident at these ages.

Children with DCD showed more deficiencies in somatosensory and motor performance test. Imitation of postures test which was used in our study assesses the somatosensory perception and praxis. These children are more affected than their healthy peers in cognitive management of motor movement, and they have problems in the spatial and temporal parameters of movement.

In the study of Schoemaker et al., they showed points to the children approximately 2 cm away from the point that they are touching in the tests of perception of the tactile localization. It may be related that these children may experience problems in visual, proprioceptive, and tactile perception and even studies assessing the perception of tactile stimuli in children with DCD are rare. Some studies reported that tactile perception and proprioception are important contributors for the development for body image which is thought to have an important role of development of praxis [24]. These results support the results of our study.

When results of the sensory integration test are checked in general, actually both groups are differences in some aspects. No difference was found between the two groups in some parameters. It was seen that children with DCD may have problems in sensory integration process.

WeeFIM assesses the activities of daily living, it rather shows the performance in the parameters related to the more personal and social development area. When it is considered that the children spend more time on play in their activities of daily living, it can be seen that WeeFIM may have missing aspect of evaluation of daily living activities of children. For this reason, evaluation of motor performance in children may give information about the activities of daily living.

The diagnosis and symptoms of DCD are more evident in school ages. Eating, dressing, and sphincter control activities are skills that are acquired in the early developmental stages. According to this reason there was no difference between the groups. On the contrary, in the study of Rosenblum [25], it is stated that the children with DCD between the ages 4–8 have difficulties in dressing and eating activities. The mean age in children with DCD was higher in our study. According to this result it is concluded that even the development in the activities of daily living is slower in DCD compared with their healthy peers, these abilities are acquired before the school age. In the study of Summers et al. [26], it is shown that after the age 8, the toileting skills are the same in children with DCD and healthy peers. When the areas of mobility and locomotion were checked, all the cases in the study were independent in their transfer and mobility, and all of their scores they were the same. In some studies in the literature it is seen that there are some deficiencies in gross motor functions of children with DCD [27,28]. However in our study, there was no difference in the transfer activities which are part of gross motor functions. WeeFIM is a test which evaluates the execution of these activities regardless the quality of the movement. Because of that reason WeeFIM can be inadequate for evaluating the motor functions in children.

Comprehension and expression social communication, problem-solving skills and the results obtained from the evaluation of memory were affected in children with DCD. As a consequence of their motor problems, they may demonstrate additional difficulties, including poor perceived competence, social isolation, low self-worth, anxiety and depressive symptoms, even at early ages [29,30]. Also according to the results of our study, although the children with DCD have not got any mental problems, they have problems in sensory integration process and they have poor motor performance. It is taught that these deficiencies accounts to some problems in the areas of comprehension, communication, social interaction, problem solving and memory according to their healthy peers.

The correlation between the Ayres Southern California Sensory Integration Tests and WeeFIM was analyzed and significant correlations were identified between kinesthesia and social communication and imitation of postures and social communication in typically developing children. When the mobility of these children at this age is considered impairments in social communication skills can be seen. They use body movements during these communication skills. Significant correlation was found in children with DCD between graphesthesia and self-care. This result suggested that a better tactile system in children with DCD the higher independence in the activities of daily living. Including the tactile and proprioceptive approaches in the treatment programs will have a positive effect on the success in rehabilitation programs.

For this reason it is thought that, after the early diagnose, tactile and proprioceptive contents should constitute an important role in the rehabilitation programme of children with developmental coordination disorder to be more independent in the activities of daily living.

It is recommended that future work addresses the use of sensory integration approaches for designing effective interventions in children with DCD to be independent in the activities of daily living. Especially in the area of perception, more detailed and extensive studies must be done.

## Key messages

1. Problems in taking visual, tactile and proprioceptive senses and integrating them in an appropriate way leads to deficiencies in activities of daily living in children with DCD.

2. A better tactile system in children with DCD the higher independence in the activities of daily living.

3. Tactile and proprioceptive contents should constitute an important role in the rehabilitation programme of children with developmental coordination disorder to be more independent in the activities of daily living.

## Competing interests

The authors declare that they have no competing interests.

## Authors' contributions

HK conceived and designed the study. BE was responsible for sociodemographic data taking, clinical examination and implementation of the sensory integration and activity of daily living tests in children with developmental coordination disorder. He analyzed the data, wrote the manuscript and took part in revision and submission. ID revised the manuscript for important intellectual content and took part in data analysis. All authors read and approved the final manuscript.
